# Intracranial Neurofeedback Modulating Neural Activity in the Mesial Temporal Lobe During Memory Encoding: A Pilot Study

**DOI:** 10.1007/s10484-023-09595-1

**Published:** 2023-07-05

**Authors:** Koji Koizumi, Naoto Kunii, Kazutaka Ueda, Kazuhiko Takabatake, Keisuke Nagata, Shigeta Fujitani, Seijiro Shimada, Masayuki Nakao

**Affiliations:** 1 Department of Mechanical Engineering, Graduate School of Engineering, The University of Tokyo, Tokyo, Japan; 2 Department of Neurosurgery, The University of Tokyo, Tokyo, Japan

**Keywords:** Neurofeedback, Memory encoding, Intracranial electrode, Mesial temporal lobe, Epilepsy

## Abstract

Removal of the mesial temporal lobe (MTL) is an established surgical procedure that leads to seizure freedom in patients with intractable MTL epilepsy; however, it carries the potential risk of memory damage. Neurofeedback (NF), which regulates brain function by converting brain activity into perceptible information and providing feedback, has attracted considerable attention in recent years for its potential as a novel complementary treatment for many neurological disorders. However, no research has attempted to artificially reorganize memory functions by applying NF before resective surgery to preserve memory functions. Thus, this study aimed (1) to construct a memory NF system that used intracranial electrodes to feedback neural activity on the language-dominant side of the MTL during memory encoding and (2) to verify whether neural activity and memory function in the MTL change with NF training. Two intractable epilepsy patients with implanted intracranial electrodes underwent at least five sessions of memory NF training to increase the theta power in the MTL. There was an increase in theta power and a decrease in fast beta and gamma powers in one of the patients in the late stage of memory NF sessions. NF signals were not correlated with memory function. Despite its limitations as a pilot study, to our best knowledge, this study is the first to report that intracranial NF may modulate neural activity in the MTL, which is involved in memory encoding. The findings provide important insights into the future development of NF systems for the artificial reorganization of memory functions.

## Introduction

Epilepsy is a major neurological disease that occurs in approximately 50 million individuals worldwide (World Health Organization, 2019). In Japan, 1% of the population (approximately 1 million people) have epilepsy, and approximately 300,000 of them have intractable epilepsy that does not respond to drug treatment. For mesial temporal lobe (MTL) epilepsy, the most common form of intractable epilepsy, resection of the hippocampus and surrounding structures has been established as a safe and effective surgical treatment. However, the MTL plays an essential role in memory encoding (Squire & Zola-Morgan, 1991), and thus, there is a risk of memory dysfunction following resection. Particularly, patients with high verbal memory and without typical hippocampal sclerosis preoperatively who undergo surgery on the language-dominant side have reduced verbal memory function, especially episodic memory (Gleissner et al., 2002; Helmstaedter et al., 1996, 2011; Hermann et al., 1994), and decreased quality of life postoperatively (Mihara et al., 1994, 1996).

Thus, there have been attempts to predict postoperative memory impairment using preoperative electroencephalogram (EEG) of the parahippocampal gyrus, electrical stimulation, and functional magnetic resonance imaging (fMRI) (Bonelli et al., 2010; Kunii et al., 2014; Tani et al., 2016). However, memory decline after surgical procedures has not been resolved.

Patients with severe mesial temporal sclerosis who develop early epileptic seizures show little change in memory function even after surgery (Trenerry et al., 1993). This difference in the degree of memory impairment after resection surgery can be explained by residual memory function in the MTL on the side to be resected, preliminary memory function in the contralateral MTL, or reorganization of function due to the transfer of memory function to the contralateral side (Chelune et al., 1991). Such reorganization of memory function may occur in the contralateral MTL (Golby et al., 2002; Richardson et al., 2003), in neocortical areas (Detre et al., 1998), and in the posterior hippocampus (Bonelli et al., 2010) on the side to be resected. If this reorganization of memory function can be artificially generated using the preoperative intracranial EEG measurement, a multidimensional therapeutic approach to refractory temporal lobe epilepsy may be completed: memory impairment prediction, memory function modification, and memory impairment avoidance.

Neurofeedback (NF), which controls brain function by converting brain activity into perceivable information and providing feedback, has been recently attracting attention. NF training alleviates the symptoms of anxiety, depression, schizophrenia (Schoenberg & David, 2014), and attention deficit hyperactivity disorder (Lofthouse et al., 2012). Most NF studies use noninvasive brain activity measurement modalities, such as fMRI (Sulzer et al., 2013), near-infrared spectroscopy (Kohl et al., 2020), and scalp EEG (Enriquez-Geppert et al., 2017). In intractable epilepsy patients, intracranial electrodes may be implanted to diagnose epileptic foci, and there is growing interest in applying this invasive brain activity measurement method to the brain–computer interface (Herff et al., 2020). Intracranial electrodes have superior temporal resolution to fMRI and are not affected by artifacts. Intracranial electrodes also allow localized access to important deeper structures such as the hippocampus, insula, and amygdala. NF efforts that take advantage of these intracranial electrodes include studies using electrocorticography (ECoG) (Gharabaghi et al., 2014), local field potentials (Khanna et al., 2017), spike rate metric (Chaudhary et al., 2022), and depth electrodes (Bichsel et al., 2021) to modulate sensorimotor function. One study (Yamin et al., 2017) attempted to modulate emotional function by acquiring gamma rhythms in the amygdala with deep electrodes and performing NF. However, these efforts have only recently begun.

Thus far, the effect of NF on memory function has been actively studied using scalp EEG (Enriquez-Geppert et al., 2014; Eschmann et al., 2020; Eschmann & Mecklinger, 2022; Reiner et al., 2014, 2018; Reis et al., 2016; Rozengurt et al., 2017; Tseng et al., 2021; Wang & Hsieh, 2013). Based on the relationship between memory function and theta rhythm (4–8 Hz) (Guderian & Düzel, 2005; Herweg et al., 2016; Hsieh & Ranganath, 2014) and given that many scalp EEG studies have reported an increase in theta power during memory tasks (Düzel et al., 2005; Hanslmayr et al., 2009; Osipova et al., 2006), most memory NF studies using scalp EEG employ NF to increase theta power. These studies have suggested that increased theta activity in the frontal midline area improves control processes in memory retrieval (Eschmann et al., 2020) and enhances memory consolidation (Reiner et al., 2014; Rozengurt et al., 2017), providing evidence on the relationship between theta rhythm and memory function. Studies using intracranial electrodes have also reported increased theta power in the MTL during successful encoding and retrieval (Fell et al., 2011; Herweg et al., 2020a; Lega et al., 2012; Lin et al., 2017; Miller et al., 2018; Sederberg et al., 2003; Solomon et al., 2019a) and increased theta phase synchronization between the hippocampus and other cortices (rhinal cortex, prefrontal cortex) during encoding (Fell et al., 2003; Gruber et al., 2018). Meanwhile, some studies have reported decreased theta power in the MTL during encoding (Ezzyat et al., 2017, 2018; Fellner et al., 2019; Greenberg et al., 2015; Kragel et al., 2017; Long & Kahana, 2015; Solomon et al., 2019b). As such, the causal relationship between the increase or decrease in theta power of MTL and memory function remains unclarified. In any case, using intracranial electrodes has great potential for advancing memory NF research as they allow direct acquisition of MTL signals. Burke et al. (2015) recently reported modulated performance of memory encoding when the neural activity of the MTL was used to adjust the stimulus presentation timing, supporting that intracranial electrodes may enhance memory function. However, to the best of our knowledge, no study has established a memory NF system using intracranial electrodes or conducted training to coordinate memory functions in the MTL.

Considering that the brain autonomously reorganizes its functions in patients with severe mesial temporal sclerosis (Bonelli et al., 2010; Chelune et al., 1991; Detre et al., 1998; Golby et al., 2002; Richardson et al., 2003), NF training, which uses the brain’s autonomous learning ability to coordinate brain activity and cognitive functions, is a promising method of artificially reorganizing memory functions in MTL. However, to the best of our knowledge, no studies have addressed the artificial reorganization of memory functions by NF using intracranial electrodes in MTL. Further, whether NFs can modulate neural activity in MTLs related to memory should first be confirmed. Thus, the present study aimed to determine whether NF training using intracranial electrodes implanted in the MTL could modulate neural activities on the verbal memory-dominant side during memory encoding. The secondary objective was to verify how verbal memory function changes as NF training progresses.

## Methods

### Study Design and Participants

This pilot study was a two-person case study and an observational study with no control condition. Two native Japanese-speaking patients with intractable epilepsy who had intracranial electrodes implanted to determine the epileptic focus at the University of Tokyo Hospital were enrolled. The language-dominant side was confirmed using functional MRI before electrode implantation or bedside cortical electrical stimulation via subdural electrodes during a language task (Kunii et al., 2011). Considering the concordance between verbal dominance and verbal memory dominance (Binder et al., 2008), we assumed that both participants’ verbal memory dominant sides were on the left side. Both participants had normal verbal memory level (Japanese version of the Wechsler Memory Test mean [standard deviation] index score: 100 [15]) (Table 1).

**Table 1:** Participant characteristics

ID	Age (years)	Age at onset (years)	MRI findings	Epileptic focus	NF target	FIQ	Language laterality	WMS-R verbal memory index
P01	50s	44	No lesion	Rt MTL	Lt MTL	71–80	Lt	92
P02	20s	20	No lesion	Rt MTL	Lt MTL	101–110	Lt	109

NF, neurofeedback; FIQ, full-scale intelligence quotient; WMS-R, Wechsler Memory Scale-Revised; Lt, left; Rt, right; MTL, medial temporal lobe

This study was approved by the institutional review board of the University of Tokyo Hospital (No.1797) and was conducted in accordance with the Declaration of Helsinki. Written informed consent was obtained.

### Electrode Localization and Data Acquisition

**Fig. 1:**
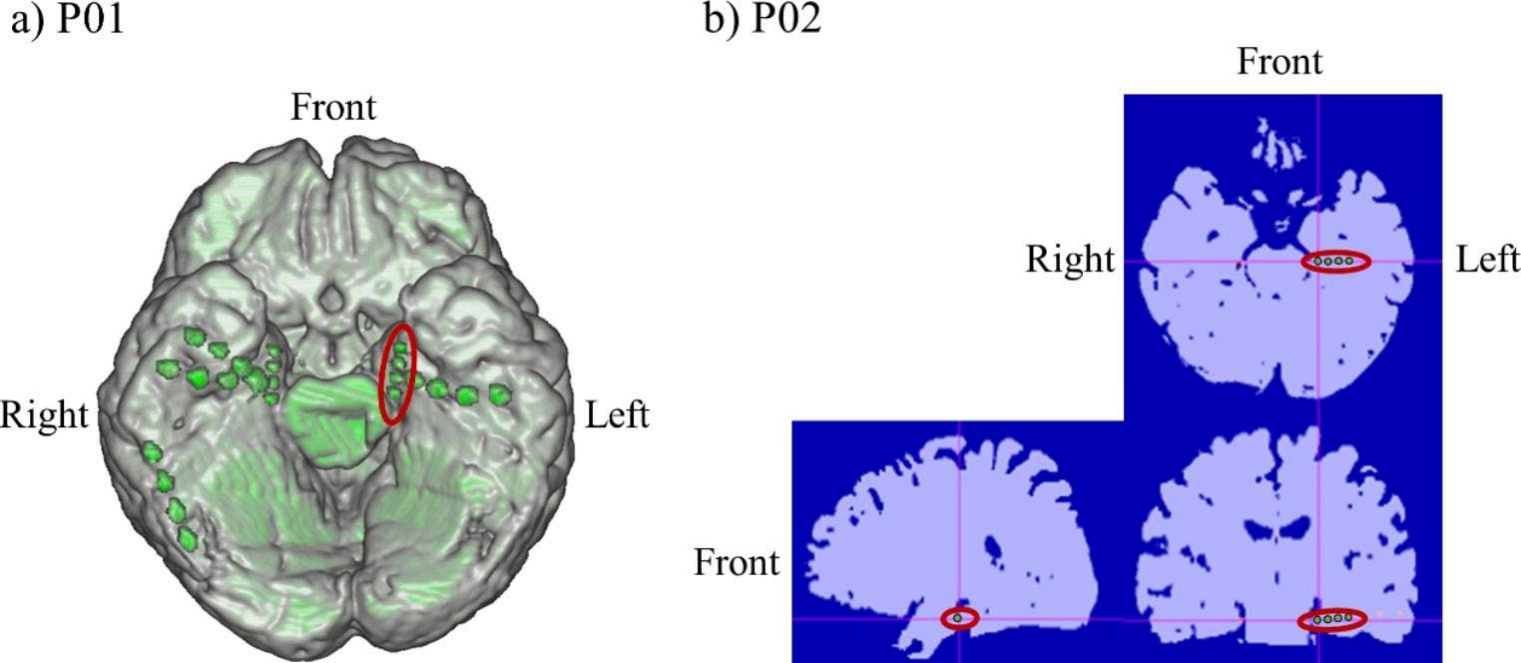
Intracranial electrodes used for neurofeedback. (**a**) P01. (**b**) P02

The location of the intracranial electrodes was determined according to the suspected epileptic foci. For participant 01 (P01), a T-shaped subdural electrode (Unique Medical, Tokyo, Japan) was placed in the left MTL, covering the parahippocampal gyrus (Fig. 1a). Four 1.5-mm-diameter platinum contacts were aligned longitudinally along the parahippocampal gyrus at 5-mm intervals (center-to-center) and were used to record intracranial EEG and NF. In P02, a depth electrode (Unique Medical, Tokyo, Japan) was placed in the left MTL (Fig. 1b). Four 1-mm-long platinum contacts were placed 5 mm apart (center-to-center) from the tip located in the hippocampus and were used to record intracranial EEG and NF. Reference electrodes were placed on the subdural side of the right parietal lobe. Intracranial EEG data were recorded at a sampling rate of 512 Hz using the gUSBamp (gTec, Schiedlberg, Austria) and band-pass filter (0.5–200 Hz). Notch filter at 50 Hz was applied to eliminate power line interference.

### Memory Neurofeedback Paradigm

**Fig. 2:**
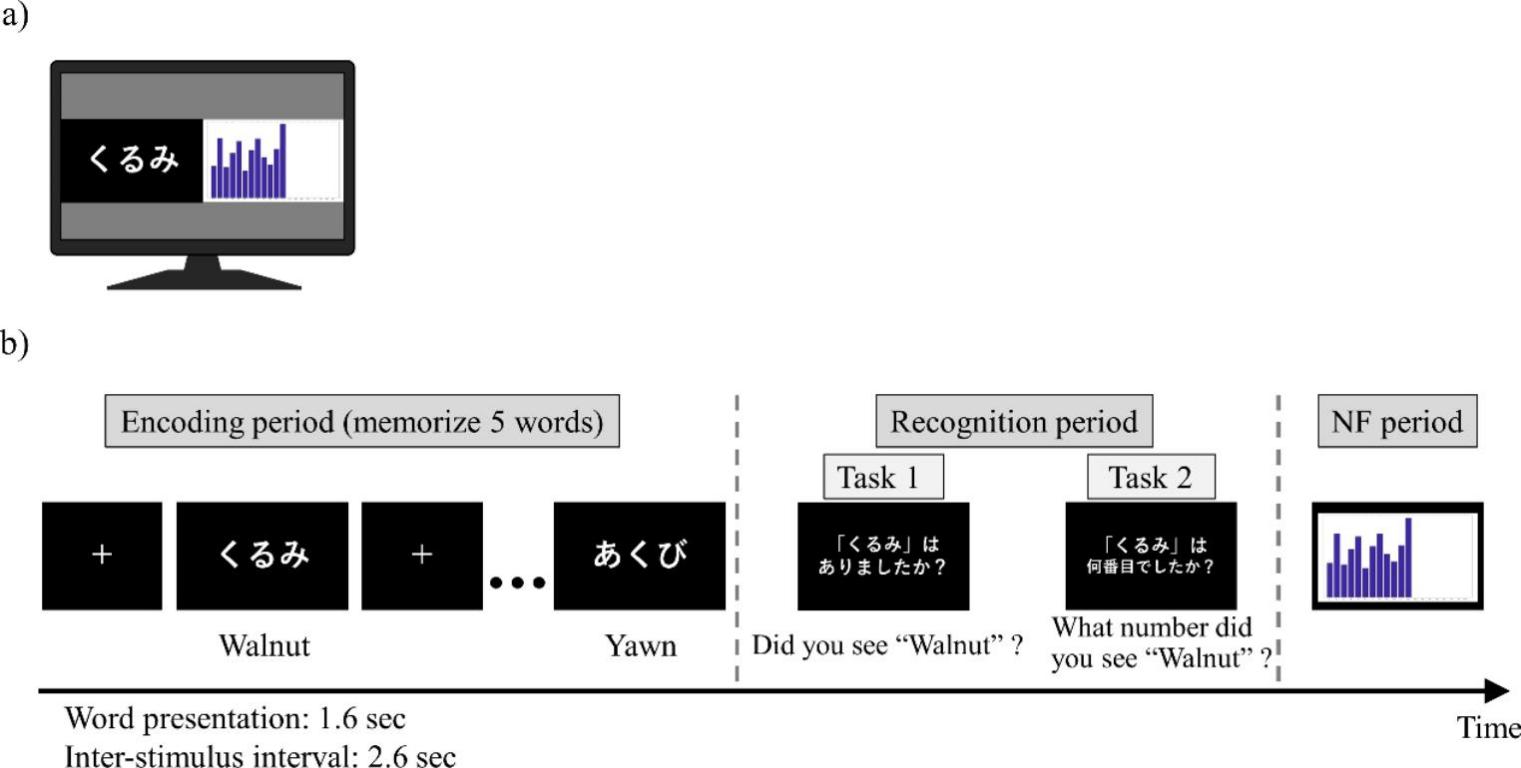
Memory neurofeedback paradigm. (**a**) Example of monitor screen during encoding period. (**b**) One-trial sequence of memory neurofeedback (NF)

The neural activity involved in memory had to be modulated; thus, the neural activity feedback had to be coordinated while performing the memory task. Therefore, we employed intermittent feedback as the NF method to reduce the risk of interference between the memory and NF processes by constructing a memory NF system in which the two processes are separate but maintain continuity. Before the experiment, participants read instructions to ensure that they fully understood the content of the task. Participants sat in an electrically shielded room and were instructed to watch a monitor screen showing the visual stimuli during the task. The left screen presented the instructional text and memory task, while the right section presented the visualization of NF (Fig. 2a).

First, for 20 s at the beginning of each session, the participants were asked to rest while looking at the fixed viewpoint displayed in the center of the left section. Each session consisted of 20 trials in the subsequent memory NF paradigm, and each trial consisted of a memory task (encoding period followed by a recognition period) and NF period (Fig. 2b). During the encoding period, five words were presented sequentially in the center of the left section for 1.6 s at 2.6-s intervals, and participants memorized them. In the subsequent recognition period, one word was presented, and the participants were asked whether they saw it (Task 1) and what number they saw it (Task 2), each for 8 s. The participants responded to these questions using a keystroke. Subsequently, in the NF period, the theta power of the left MTL in the encoding period was visualized on the right screen as the height of a new bar with those of all previous trials for 10 s. The duration per session was set at approximately 15 min, considering the patient’s physical and mental fatigue. The memory task and the NF are described in more detail below.

#### Memory Task

The words prepared for the memory task were chosen from the Word List by Semantic Principles version 1.0 provided by the Center for Language Resource Development, National Institute for Japanese Language and Linguistics (2019). This word database contains 96,557 words with familiarity estimated using crowdsourcing and Bayesian linear mixed models (Asahara, 2019). First, Japanese hiragana or katakana words consisting of three letters or three syllables were extracted to create many six-word word sets. The word combinations were adjusted such that the average familiarity scores of the six words were equal across sets. Twenty word sets were extracted per session, with one word set per trial. Five of the six words in the word set were presented during the encoding period. In the subsequent recognition period, one of the five words presented in the encoding period was presented as an old stimulus or the remaining word not presented in the encoding period was presented as a new stimulus. Of the 20 trials, 10 trials were set up to present the old stimulus, and 10 trials, the new stimulus. The sequence of trials that presented either old or new stimuli was randomized. Only in session 2 of P02 did a system error occur, and the memory NF was terminated at trial 17.

For recognition task 1, the accuracy score (the probability of correctly answering whether the stimulus was old or new out of all responses) and recall score (the probability of correctly answering an old stimulus as an old stimulus) were calculated. For recognition task 2, the accuracy score (i.e., the probability of correctly answering the order by which the old stimulus in task 1 was presented) was calculated. For example, in the trial shown in Fig. 2b, “walnut” was the first of the five words presented, so the correct answer for task 2 is “first.“

#### Neurofeedback

Real-time analysis of the intracranial EEG data and feedback of the analysis results were performed using the original program created using Simulink-based application, g.HIsys (gTec, Schiedlberg, Austria) and MATLAB R2020a (MathWorks, Natick, MA, USA). In the real-time analysis of P01, intracranial EEG data were extracted for 13 s ((1.0 + 1.6) s × 5 words) during the encoding period. Theta power (4–8 Hz) was calculated for each electrode and averaged across the electrodes. In the real-time analysis of P02, artifacts due to epileptic spike waves were reduced after extracting intracranial EEG data. In this process, artifact subspace reconstruction (ASR, EEGLAB function “clean_rawdata.m,” (Chang et al., 2018; Mullen et al., 2015)) was conducted with parameter k set to 4. Then, theta power was calculated using 12 s of data, excluding the first 1 s before the first word was presented. Theta power was visualized as bar height. We explained to the participants that brain activity during the encoding period was visualized as a bar and that they would be asked to try to get the bar as high as possible through trial and error. To allow the participants to make trial-and-error adjustments to the bar height, the bar was presented together with those of all previous trials so that the relative heights could be checked. The participants were asked to concentrate on the task during the encoding period and did not provide explicit instructions regarding mental strategies to control brain activity.

### Offline Data Analysis

After extracting the data for the encoding period, the value of the ASR parameter (k) was adjusted to reduce artifacts according to the degree of epileptic spike-wave contamination. Specifically, waveform correction by ASR was based on the percentage of EEG potentials exceeding the 13-second potential mean ± 3 standard deviations: k = 10 for < 1%, k = 6 for 1–1.5%, and k = 4 for > 1.5%. The 12s data were then extracted for each trial, excluding the first 1 s before the first word was presented.

The power spectral density (PSD) of intracranial EEG was calculated for each trial using the Welch periodogram method (1-second Hanning window, 50% overlapping) and averaged among electrodes. To examine the characteristics of PSD related to memory encoding, the average PSDs in correct trials and those in error trials in the recognition task were calculated for each session, excluding sessions in which all trials were answered correctly. The averaged PSDs of correct and error trials were transformed into a 10 × log10 (µV^2^/Hz) scale and compared using paired t-tests (corrected for multiple comparisons by the number of frequency bins, Holm-Bonferroni method, α = 0.05). In addition, to examine the changes in PSD with NF training, the PSDs between the first and final session were compared using the Wilcoxon rank sum test (corrected for multiple comparisons by the number of frequency bins, Holm-Bonferroni method, α = 0.05).

Theta power (4–8 Hz) was calculated for each trial and each electrode by integrating the PSD estimate and then averaging across the electrodes. Finally, theta powers were transformed into a logarithmic scale and examined for changes across the sessions. One-way analysis of variance (ANOVA) with post-hoc Tukey’s test was used to compare theta power between sessions (α = 0.05). To explore the correlation between memory function and the NF signal, we performed three correlational analyses (Spearman’s rank order correlation, corrected for multiple comparisons using the Holm-Bonferroni method, α = 0.05), focusing on the change in memory task performance and the NF signal between sessions. The mean theta power of all trials was used for correlation analysis with the accuracy score in recognition Task 1. For the correlation analysis with the recall score in Recognition Task 1, the mean theta power of all trials in which the old stimuli were presented in Recognition Task 1 was used. For the correlation analysis with the accuracy score in Recognition Task 2, the mean theta power of trials that correctly responded to the old stimulus as the old stimulus in Recognition Task 1 was used.

## Results

### Patient Response

P01 and P02 completed six and five memory NF sessions, respectively. No response was obtained from P01 regarding mental strategies during the NF. P02, meanwhile, adopted the strategy of making associations between presented words. After session 3, P02 responded that bar height increased when word associations were made, especially when good word associations were made, while bar height decreased when word associations were made forcibly.

### Comparison of Power Spectral Density

**Fig. 3:**
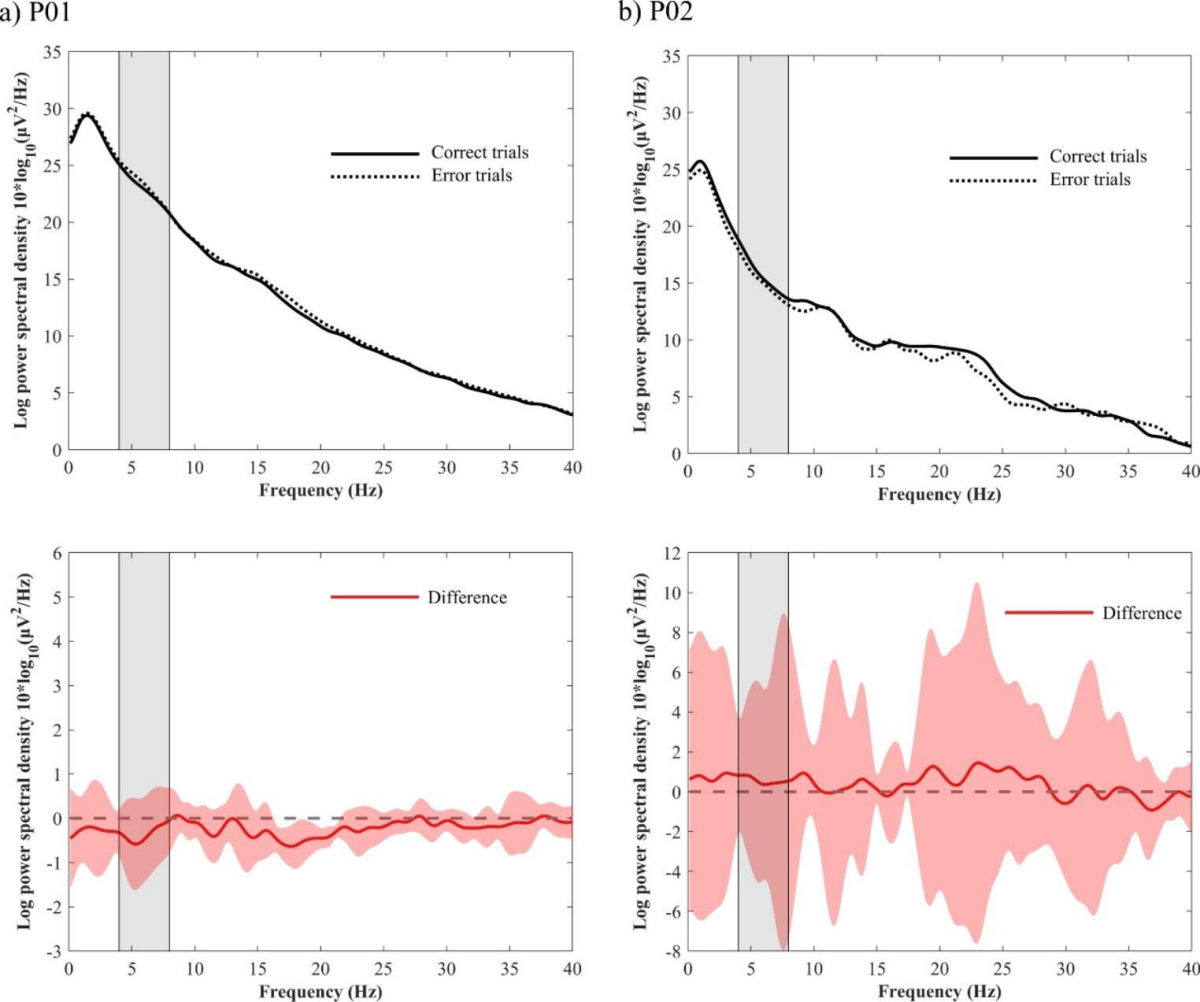
Differences in power spectral density (correct trials − error trials. (**a**) P01. (**b**) P02). Solid and dotted black lines indicate the mean power spectral density (PSD) of correct trials (solid line) and error trials (dotted line), respectively (upper panel). Red line indicates the mean differences of PSDs (lower panel). The red shaded area around the solid red line indicates the 95% confidence interval. The light gray zone indicates the theta frequency range (4–8 Hz)

Given that the accuracy/recall score of recognition task 1 exceeded 80% for both participants (Table 2) and the number of error trials was insufficient for PSD comparison with correct trials, comparisons were made based on the performance of recognition task 2. In P01, the mean PSDs for correct trials were lower than those for error trials across frequencies, with a difference peak observed in the theta band. However, no significant differences were observed at any of the frequencies. At P02, there were no significant differences at any frequency, and the confidence intervals were large (Fig. 3).

**Table 2:** Recognition tasks and correlation with theta power

ID	Recognition task		Session						Correlation
			1	2	3	4	5	6	
P01	Task 1	Accuracy (%)	90	90	100	90	100	100	*r* = 0.49, *p* = 0.33
		Recall (%)	90	80	100	80	100	100	*r* = 0.80, *p* = 0.06
	Task 2	Accuracy (%)	78	50	60	75	70	70	*r* = 0.35, *p* = 0.50
P02	Task 1	Accuracy (%)	100	100	100	100	95		*r* = − 0.35, *p* = 0.56
		Recall (%)	100	100	100	100	90		*r* = − 0.35, *p* = 0.56
	Task 2	Accuracy (%)	90	100	100	90	78		*r* = − 0.53, *p* = 0.36

**Fig. 4:**
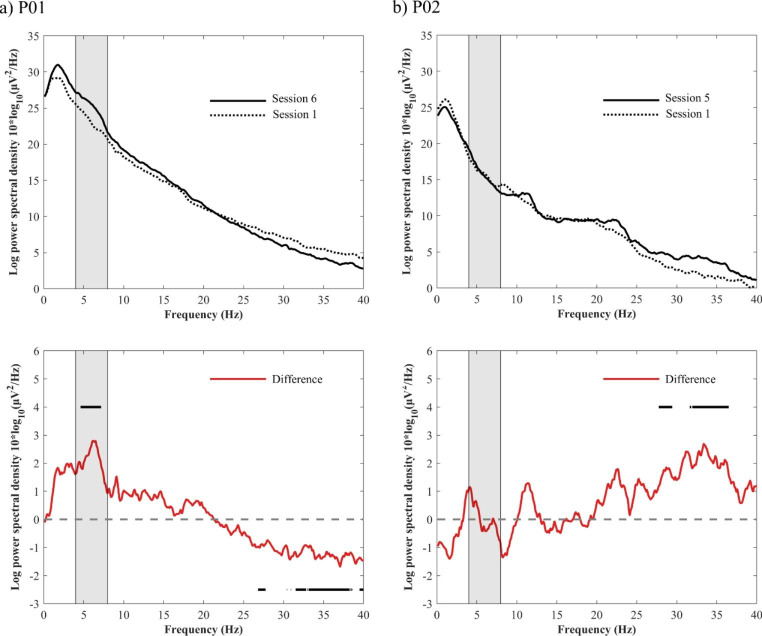
Changes in power spectral density (the final session – session 1). (**a**) P01. (**b**) P02). Solid and dotted black lines indicate the median power spectral density (PSD) in the final session (solid line) and in session 1 (dotted line), respectively (upper panel). Red line indicates the median differences of PSDs (lower panel). Black bars above and below the lines indicate the frequency range that is significantly higher and lower in PSD in the final session than in the first session (*p* < 0.05, corrected for multiple comparisons by the number of frequency bins, Holm-Bonferroni method). The light gray zone indicates the theta frequency range (4–8 Hz)

In P01, the PSD of the final session was significantly higher in the theta range and significantly lower in the fast beta and gamma range than in session 1 (*ps* < 0.05, corrected for multiple comparisons by the number of frequency bins, Holm-Bonferroni method). In contrast, in P02, the PSD of the final session was significantly higher in the fast beta and gamma range than in session 1 (*ps* < 0.05, corrected for multiple comparisons by the number of frequency bins, Holm-Bonferroni method) (Fig. 4).

### Neurofeedback Regulation Based on the Feedback Signal

**Fig. 5:**
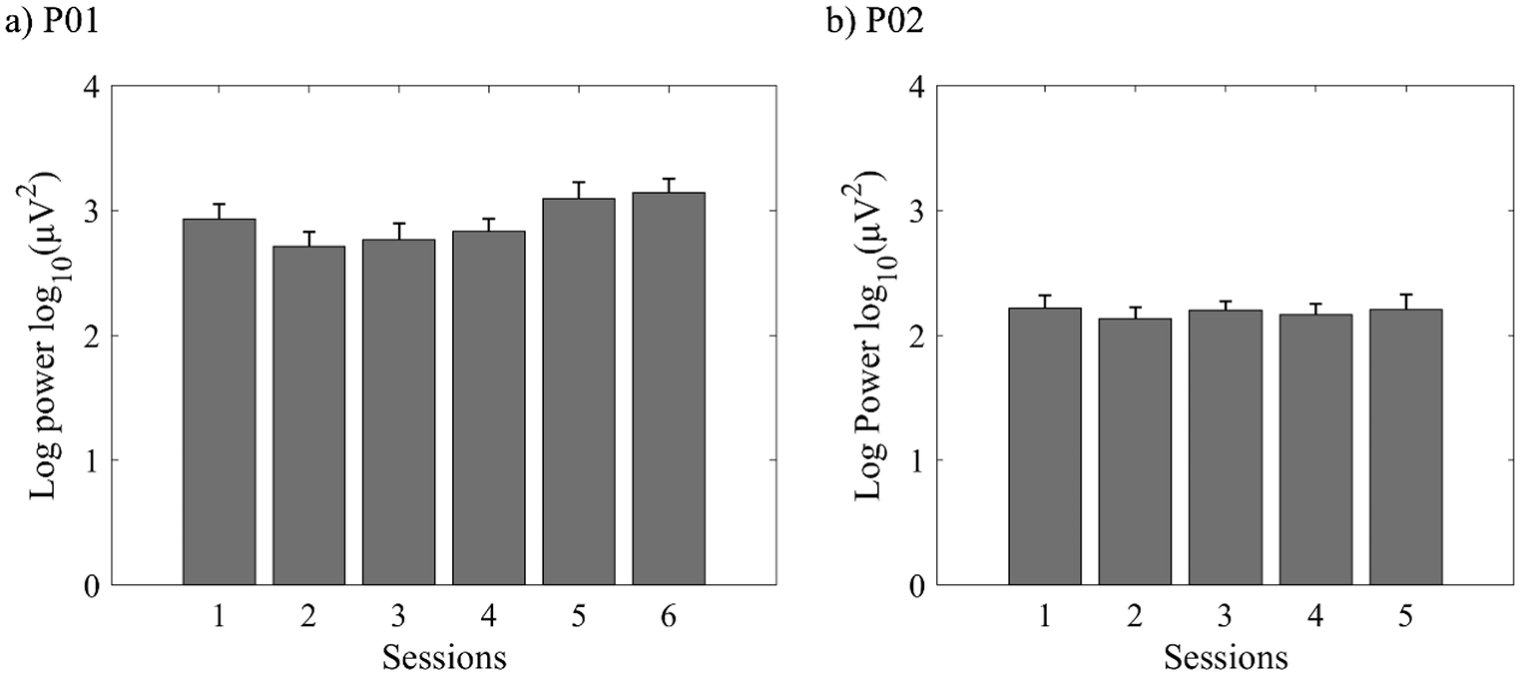
Theta power changes over sessions. (**a**) P01. (**b**) P02. Error bars indicate one standard deviation of the mean

One-way ANOVA showed significant differences between sessions for P01 (*F*(5, 114) = 44.865, *η*^*2*^ = 0.663, *p* < 0.001). The post-hoc Tukey test showed that theta power was significantly lower in sessions 2 and 3 than in session 1 (*ps* < 0.01) and significantly higher in the later stage (sessions 5 and 6) than in the early (sessions 1 and 2) and middle stages (sessions 3 and 4) (*ps* < 0.01). In contrast, in P02, there was no significant differences between the sessions (*F*(4, 92) = 2.258, *η*^*2*^ = 0.089, *p* = 0.069) (Fig. 5).

### Behavioral Outcome and Correlation with Feedback Signal

Table 2 summarizes the results of the changes in performance of the recognition tasks and their correlation with theta power in the left MTL. In Task 1, the accuracy scores were more than 90%, and the recall scores were more than 80% in all sessions for both participants. The scores of Task 2 were lower than those of Task 1, and the results varied among the sessions. No significant correlation was found between recognition task scores and theta power of the left MTL.

## Discussion

The present study suggests that memory NF for MTL may modulate not only theta-band power, but also high-frequency band power. Theta power was significantly increased while high-frequency power (fast beta and gamma) was significantly decreased after more than five sessions of NF training in one (P01) of two participants with intractable epilepsy. In addition, in P01, the PSD of the left MTL during memory encoding was lower in the correct trials than in the error trials, especially in the theta band, although this was not significant.

Decreases in theta power during episodic memory processing, especially successful encoding, have often been reported in intracranial studies in humans (Burke et al., 2013; Ezzyat et al., 2017, 2018; Fellner et al., 2019; Greenberg et al., 2015; Herweg et al., 2020b; Kragel et al., 2017; Long et al., 2014; Long and Kahana, 2015; Sederberg et al., 2007; Solomon et al., 2019). The results for P01 are in line with those of previous studies. The decrease in theta power at encoding in intracranial studies, including the present study, is inconsistent with the findings of scalp EEG studies, which are characterized by an increase in theta power (Düzel et al., 2005; Hanslmayr et al., 2009; Osipova et al., 2006). This discrepancy between scalp EEG and intracranial electrode studies is most likely due to the difference in the spatial resolution of the EEG measurement methods (Herweg et al., 2020b). Herweg et al. (2020b) suggested that intracranial electrodes recorded a decrease in theta power in localized areas, but the oscillations were highly correlated between electrodes; meanwhile, scalp electrodes observed this synchronization as a relative increase in theta power. As such, intracranial electrodes, which can acquire signals directly from the MTL, are more useful than scalp EEG in modifying memory functions in the MTL located deep in the brain.

After conducting memory NF training for P01, we observed an increase in theta power and decrease in the high-frequency band, consistent with previous findings (Greenberg et al., 2015; Long & Kahana, 2015; Solomon et al., 2019b). Increased coupling between the theta rhythm phase and the amplitude of high-frequency rhythm in the MTL, especially in the hippocampus, during episodic memory encoding has also been reported (Lega et al., 2016; Mormann et al., 2005), suggesting the importance of interaction between these two frequency bands in memory formation.

Interestingly, the increased theta power in P01 occurred during the late NF training stage. This could be because the intermittent feedback method is employed in neurofeedback. The usefulness of intermittent feedback has been reported in several studies (Emmert et al., 2017; Hellrung et al., 2018; Johnson et al., 2012; Zilverstand et al., 2015). In a previous study, intermittent feedback took longer to learn than continuous feedback when participants can directly influence the feedback signal through simple behavioral strategy choices (Oblak et al., 2017). Continuous feedback may allow for faster and more efficient learning of memory functions. The risk of interference between memory tasks and continuous NF can be decreased by adjusting the background color of the screen presenting the memory task according to the power of theta (Monseigne et al., 2019) or by visually presenting the memory task while aurally presenting NF using music (Takabatake et al., 2021).

The different result for P02 can be explained as follows. The first was the difference in the difficulty of the memory task for each participant. P02 had a smaller number of sessions (only three) for comparing PSDs in the correct-and-error trials. Thus, the confidence intervals were large, and it was more difficult to observe the difference between the correct and incorrect trials than in P01. The second is the difference in the electrodes used for NF: P01 used a subdural electrode, whereas P02 used a depth electrode. Compared with depth electrodes, subdural electrodes are implanted to cover the cortical area and acquire signals from a broader area of the targeted region (Herff et al., 2020; Parvizi & Kastner, 2018). In addition, the four platinum contacts used in P02 were aligned at 5-mm intervals from the tip located in the hippocampus toward the lateral temporal lobe, which may have been more affected by other brain region activity than subdural electrodes. The impact of different electrodes used on the NF effect needs to be examined in the future. The third is the effect of the mental strategy employed by the P02. P02 started to remember by relating words displayed in the middle stage. Increased high-frequency power in the hippocampus in word memory encoding has been suggested to be related to semantic processing (Sederberg et al., 2007) and the process of linking words to context (Long & Kahana, 2015). Compared to session 1, the final session of P02 may have had significantly increased high-frequency PSD during encoding using the mental strategy of word association.

Interestingly, P02 stated that bar height increased when word associations were made, especially when good word associations were made, while bar height was decreased when word associations were made forcibly. The use of semantic encoding strategies and episodic (temporal) encoding strategies have a trade-off relationship (Golomb et al., 2008; Healey et al., 2014; Sederberg et al., 2010). Differences in EEG rhythm depend on whether a semantic encoding strategy is used and on the context during encoding (Hanslmayr et al., 2009; Staudigl & Hanslmayr, 2013). A recent study reported an increase in theta power in the left hippocampus when recalling words with a close semantic distance and a decrease in theta power when recalling words with distant semantic distance (Solomon, Lega et al., 2019). A good word association can be interpreted as a state in which a closer semantic relationship is found between words than when word associations are made forcibly. As such, the increase in relative theta power due to the shift from temporal to semantic encoding strategies and due to good word associations may be reasonable.

A recent study (Chaudhary et al., 2022) used intracranial microelectrodes, like our study, at the premotor cortex in one patient and reported a highly successful neural control of cellular neural activity in a NF task over many sessions with immediate NF control after introduction of the task. The differences between their results and ours can be explained based on several differences in the NF methods employed. First, they analyzed NF data from day 123 after electrode implantation in patients and described it in their paper. However, they first employed NF on the 86th day, and from day 106 onwards, they had already reached a level of letter selection (see their “Results section”). Since our study reports results from only a few days after the first use of NF, it is reasonable to expect differences between the studies. A more rigorous comparison of NF effects across studies would require a similar number of training days. However, due to time constraints regarding the number of days electrodes can be implanted in patients, it is practically difficult to obtain more extended sessions than those reported in this study. Second, they employed continuous feedback. Continuous feedback is expected to be faster and more efficient in learning than the intermittent feedback we employed (Oblak et al., 2017). Third, they identified the target feedback region as the sensory-motor cortex. The sensory-motor cortex is located on the brain surface and has been the target of motor-imagery brain-computer interface and other intracranial EEG studies. Many studies have suggested the possibility of modulating neural activity of this region (Bichsel et al., 2021; Gharabaghi et al., 2014; Khanna et al., 2017). On the other hand, as indicated by the fact that our study is the first to attempt NF with intracranial EEG in the medial temporal lobe, the modulability of neural activity in the MTL has rarely been discussed, and we are only now beginning to accumulate knowledge. We believe that our results will serve as a starting point for further research to investigate the possibility of memory-related neuronal activity regulation in the MTL. Finally, in their study, NF was used as an alternative method of communication other than speech for patients with ALS, and there is a difference in the participants’ motivation for NF compared to our study, which aimed to evaluate the possibility of modulating neural activity in the MTL. Studies have reported differences in motivation as a factor influencing neurofeedback success (Kadosh & Staunton, 2019).

An early NF study in cats reported successful modulation of the sensory-motor rhythm (Sterman et al., 1969). Studies in curarized rats also suggested the possibility of self-control of autonomic nervous system activity by biofeedback (Dworkin & Miller, 1986; Miller, 1969). Compared to these animal studies, human studies sometimes report difficulties in the self-regulation of neural activity, as in the present study. One possible interpretation of this factor is that there may be differences in rewards, which are positive reinforcers in learning. In animal studies, water, food, or electrical stimulation of the pleasure center of the brain are direct rewards for conditioning. NF is also based on the principle of operant conditioning (Sherlin et al., 2011), and in a typical NF study, the achievement of changing the size of the circle or adjusting the height of the bar successfully is assumed to be an indirect reward (Collura, 2014). The present study also followed the traditional feedback method, but it may not have been sufficient to function as a reward that would facilitate learning for the participants. More explicit rewards in NF learning including games (Friedrich et al., 2014), monetary rewards (Bray et al., 2007), and social rewards (Mathiak et al., 2015) have been proposed; memory-NF systems incorporating such rewards could be developed.

This study has some limitations as in both participants, there was no correlation between changes in neural activity and changes in memory function. First, based on several memory studies (Düzel et al., 2005; Guderian & Düzel, 2005; Hanslmayr et al., 2009; Herweg et al., 2016; Herweg et al., 2020b; Hsieh and Ranganath, 2014), theta (4–8 Hz) was set as the frequency band targeted by memory NFs, and other frequency bands were not targeted. Some studies have reported that hippocampal activity in the low-theta band, below 4 Hz, is essential for human memory encoding (Miller et al., 2018; Solomon et al., 2019a; Staudigl & Hanslmayr, 2013). Further research is needed to evaluate the effects of different targeted frequency bands on memory NF. Second, it was difficult to recruit participants who had implanted electrodes in the MTL and could participate in NF training within a limited time frame. Therefore, this study was limited by the different types of electrodes, small sample size, small number of sessions, and inability to set a control condition. The results need to be further validated by recruiting a larger number of participants and establishing a control condition. Third, the participants were required to perform monotonous tasks throughout the assignment, which may have resulted in increased fatigue and drowsiness and decreased motivation. The importance of attention, mood, and motivation in effective learning and success in NF training has been noted (Kadosh & Staunton, 2019), and it is important to consider updating the modality from the traditional one (bar height adjustment) to one that incorporates the rewarding and gaming aspects in future studies.

Nevertheless, to our best knowledge, no studies have addressed the artificial reorganization of memory function by NF using intracranial electrodes in the MTL, and this study is the first to suggest that neural activity in the MTL, related to verbal memory function, can be modulated by NF training using intracranial electrodes.

## Conclusion

NF using intracranial electrodes may modulate neural activity in the MTL, which is involved in memory encoding. These findings extend previous findings in human memory research and also provide important insights into the future development of NF systems for the artificial reorganization of memory functions.

## Data Availability

Due to the nature of this research, participants of this study did not agree for their data to be shared publicly, so supporting data is not available.

## References

[CR2] Asahara, M. (2019). Word Familiarity Rate Estimation using a bayesian Linear mixed Model. *Proceedings of the First Workshop on Aggregating and Analysing Crowdsourced Annotations for NLP Hong Kong*, 6–14. 10.18653/v1/D19-5902

[CR3] Bichsel O, Stieglitz LH, Oertel MF, Baumann CR, Gassert R, Imbach LL (2021). Deep brain electrical neurofeedback allows Parkinson patients to control pathological oscillations and quicken movements. Scientific Reports.

[CR4] Binder JR, Sabsevitz DS, Swanson SJ, Hammeke TA, Raghavan M, Mueller WM (2008). Use of preoperative functional MRI to predict verbal memory decline after temporal lobe epilepsy surgery. Epilepsia.

[CR5] Bonelli SB, Powell RHW, Yogarajah M, Samson RS, Symms MR, Thompson PJ, Koepp MJ, Duncan JS (2010). Imaging memory in temporal lobe epilepsy: Predicting the effects of temporal lobe resection. Brain.

[CR6] Bray S, Shimojo S, O’Doherty JP (2007). Direct instrumental conditioning of neural activity using functional magnetic resonance imaging-derived reward feedback. Journal of Neuroscience.

[CR8] Burke JF, Zaghlou KA, Jacobs J, Williams RB, Sperling MR, Sharan AD, Kahana MJ (2013). Synchronous and asynchronous Theta and Gamma Activity during episodic memory formation. Journal of Neuroscience.

[CR7] Burke JF, Merkow MB, Jacobs J, Kahana MJ, Zaghloul KA (2015). Brain computer interface to enhance episodic memory in human participants. Frontiers in Human Neuroscience.

[CR9] Chang, C. Y., Hsu, S. H., Pion-Tonachini, L., & Jung, T. P. (2018). Evaluation of Artifact Subspace Reconstruction for Automatic EEG Artifact Removal. *Proceedings of the Annual International Conference of the IEEE Engineering in Medicine and Biology Society*, 1242–1245. 10.1109/EMBC.2018.8512547 10.1109/EMBC.2018.8512547 30440615

[CR10] Chaudhary U, Vlachos I, Zimmermann JB, Espinosa A, Tonin A, Jaramillo-Gonzalez A, Khalili-Ardali M, Topka H, Lehmberg J, Friehs GM, Woodtli A, Donoghue JP, Birbaumer N (2022). Spelling interface using intracortical signals in a completely locked-in patient enabled via auditory neurofeedback training. Nature Communications.

[CR11] Chelune GJ, Naugle RI, Lüders H, Awad IA (1991). Prediction of cognitive change as a function of preoperative ability status among temporal lobectomy patients seen at 6-month follow‐up. Neurology.

[CR12] Collura TF (2014). Overview. In *technical foundations of Neurofeedback*. Taylor and Francis.

[CR13] Detre JA, Maccotta L, King D, Alsop DC, Glosser G, D’Esposito M, Zarahn E, Aguirre GK, French JA (1998). Functional MRI lateralization of memory in temporal lobe epilepsy. Neurology.

[CR14] Düzel E, Neufang M, Heinze HJ (2005). The Oscillatory Dynamics of Recognition Memory and its relationship to event-related responses. Cerebral Cortex.

[CR15] Dworkin BR, Miller NE (1986). Failure to replicate visceral learning in the Acute Curarized Rat Preparation. Behavioral Neuroscience.

[CR16] Emmert K, Kopel R, Koush Y, Maire R, Senn P, van de Ville D, Haller S (2017). Continuous vs. intermittent neurofeedback to regulate auditory cortex activity of tinnitus patients using real-time fMRI - A pilot study. NeuroImage: Clinical.

[CR17] Enriquez-Geppert S, Huster RJ, Figge C, Herrmann CS (2014). Self-regulation of frontal-midline theta facilitates memory updating and mental set shifting. Frontiers in Behavioral Neuroscience.

[CR18] Enriquez-Geppert S, Huster RJ, Herrmann CS (2017). EEG-neurofeedback as a tool to modulate cognition and behavior: A review tutorial. Frontiers in Human Neuroscience.

[CR20] Eschmann KCJ, Mecklinger A (2022). Improving cognitive control: Is theta neurofeedback training associated with proactive rather than reactive control enhancement?. Psychophysiology.

[CR19] Eschmann KCJ, Bader R, Mecklinger A (2020). Improving episodic memory: Frontal-midline theta neurofeedback training increases source memory performance. Neuroimage.

[CR21] Ezzyat Y, Kragel JE, Burke JF, Levy DF, Lyalenko A, Wanda P, O’Sullivan L, Hurley KB, Busygin S, Pedisich I, Sperling MR, Worrell GA, Kucewicz MT, Davis KA, Lucas TH, Inman CS, Lega BC, Jobst BC, Sheth SA, Kahana MJ (2017). Direct brain stimulation modulates Encoding States and Memory performance in humans. Current Biology.

[CR22] Ezzyat Y, Wanda PA, Levy DF, Kadel A, Aka A, Pedisich I, Sperling MR, Sharan AD, Lega BC, Burks A, Gross RE, Inman CS, Jobst BC, Gorenstein MA, Davis KA, Worrell GA, Kucewicz MT, Stein JM, Gorniak R, Kahana MJ (2018). Closed-loop stimulation of temporal cortex rescues functional networks and improves memory. Nature Communications.

[CR23] Fell J, Klaver P, Elfadil H, Schaller C, Elger CE, Fernández G (2003). Rhinal–hippocampal theta coherence during declarative memory formation: Interaction with gamma synchronization?. European Journal of Neuroscience.

[CR24] Fell J, Ludowig E, Staresina BP, Wagner T, Kranz T, Elger CE, Axmacher N (2011). Medial temporal Theta/Alpha Power Enhancement precedes successful memory encoding: Evidence based on intracranial EEG. Journal of Neuroscience.

[CR25] Fellner MC, Gollwitzer S, Rampp S, Kreiselmeyr G, Bush D, Diehl B, Axmacher N, Hamer H, Hanslmayr S (2019). Spectral fingerprints or spectral tilt? Evidence for distinct oscillatory signatures of memory formation. PLOS Biology.

[CR26] Friedrich EVC, Suttie N, Sivanathan A, Lim T, Louchart S, Pineda JA (2014). Brain-computer interface game applications for combined neurofeedback and biofeedback treatment for children on the autism spectrum. Frontiers in Neuroengineering.

[CR27] Gharabaghi, A., Naros, G., Khademi, F., Jesser, J., Spüler, M., Walter, A., Bogdan, M., Rosenstiel, W., & Birbaumer, N. (2014). Learned self-regulation of the lesioned brain with epidural electrocorticography. *Frontiers in Behavioral Neuroscience*, *8*(429), 10.3389/fnbeh.2014.00429 10.3389/fnbeh.2014.00429 PMC4260503 25538591

[CR28] Gleissner U, Helmstaedter C, Schramm J, Elger CE (2002). Memory outcome after selective amygdalohippocampectomy: A study in 140 patients with temporal lobe Epilepsy. Epilepsia.

[CR29] Golby AJ, Poldrack RA, Illes J, Chen D, Desmond JE, Gabrieli JDE (2002). Memory lateralization in medial temporal lobe Epilepsy assessed by functional MRI. Epilepsia.

[CR30] Golomb JD, Peelle JE, Addis KM, Kahana MJ, Wingfield A (2008). Effects of adult aging on utilization of temporal and semantic associations during free and serial recall. Memory & Cognition.

[CR31] Greenberg JA, Burke JF, Haque R, Kahana MJ, Zaghloul KA (2015). Decreases in theta and increases in high frequency activity underlie associative memory encoding. Neuroimage.

[CR32] Gruber MJ, Hsieh LT, Staresina BP, Elger CE, Fell J, Axmacher N, Ranganath C (2018). Theta Phase synchronization between the human Hippocampus and Prefrontal Cortex increases during encoding of unexpected information: A Case Study. Journal of Cognitive Neuroscience.

[CR33] Guderian S, Düzel E (2005). Induced theta oscillations mediate large-scale synchrony with mediotemporal areas during recollection in humans. Hippocampus.

[CR34] Hanslmayr S, Spitzer B, Bäuml KH (2009). Brain oscillations dissociate between semantic and nonsemantic encoding of episodic Memories. Cerebral Cortex.

[CR35] Healey MK, Crutchley P, Kahana MJ (2014). Individual differences in memory search and their relation to intelligence. Journal of Experimental Psychology General.

[CR36] Hellrung L, Dietrich A, Hollmann M, Pleger B, Kalberlah C, Roggenhofer E, Villringer A, Horstmann A (2018). Intermittent compared to continuous real-time fMRI neurofeedback boosts control over amygdala activation. Neuroimage.

[CR37] Helmstaedter C, Elger CE, Hufnagel A, Zentner J, Schramm J (1996). Different effects of left anterior temporal lobectomy, selective amygdalohippocampectomy, and temporal cortical lesionectomy on verbal learning, memory, and recognition. Journal of Epilepsy.

[CR38] Helmstaedter C, Petzold I, Bien CG (2011). The cognitive consequence of resecting nonlesional tissues in epilepsy surgery—results from MRI- and histopathology-negative patients with temporal lobe epilepsy. Epilepsia.

[CR39] Herff C, Krusienski DJ, Kubben P (2020). The potential of Stereotactic-EEG for brain-computer interfaces: Current progress and future directions. Frontiers in Neuroscience.

[CR40] Hermann BP, Wyler AR, Somes G, Dohan FC, Berry AD, Clement L (1994). Declarative memory following anterior temporal lobectomy in humans. Behavioral Neuroscience.

[CR41] Herweg NA, Apitz T, Leicht G, Mulert C, Fuentemilla L, Bunzeck N (2016). Theta-Alpha Oscillations bind the Hippocampus, Prefrontal Cortex, and striatum during recollection: Evidence from simultaneous EEG–fMRI. Journal of Neuroscience.

[CR42] Herweg NA, Sharan AD, Sperling MR, Brandt A, Schulze-Bonhage A, Kahana MJ (2020). Reactivated spatial Context Guides Episodic Recall. Journal of Neuroscience.

[CR43] Herweg NA, Solomon EA, Kahana MJ (2020). Theta Oscillations in Human Memory. Trends in Cognitive Sciences.

[CR44] Hsieh LT, Ranganath C (2014). Frontal midline theta oscillations during working memory maintenance and episodic encoding and retrieval. Neuroimage.

[CR45] Johnson KA, Hartwell K, Lematty T, Borckardt J, Morgan PS, Govindarajan K, Brady K, George MS (2012). Intermittent “Real-time” fMRI feedback is Superior to continuous presentation for a Motor Imagery Task: A pilot study. Journal of Neuroimaging.

[CR46] Kadosh KC, Staunton G (2019). A systematic review of the psychological factors that influence neurofeedback learning outcomes. Neuroimage.

[CR47] Khanna P, Swann NC, de Hemptinne C, Miocinovic S, Miller A, Starr PA, Carmena JM (2017). Neurofeedback Control in Parkinsonian Patients using Electrocorticography signals accessed wirelessly with a chronic, fully implanted device. IEEE Transactions on Neural Systems and Rehabilitation Engineering.

[CR48] Kohl, S. H., Mehler, D. M. A., Lührs, M., Thibault, R. T., Konrad, K., & Sorger, B. (2020). The Potential of Functional Near-Infrared Spectroscopy-Based Neurofeedback—A Systematic Review and Recommendations for Best Practice. *Frontiers in Neuroscience*, *14*. 10.3389/FNINS.2020.00594 10.3389/fnins.2020.00594 PMC7396619 32848528

[CR49] Kragel JE, Ezzyat Y, Sperling MR, Gorniak R, Worrell GA, Berry BM, Inman C, Lin JJ, Davis KA, Das SR, Stein JM, Jobst BC, Zaghloul KA, Sheth SA, Rizzuto DS, Kahana MJ (2017). Similar patterns of neural activity predict memory function during encoding and retrieval. Neuroimage.

[CR50] Kunii N, Kamada K, Ota T, Kawai K, Saito N (2011). A detailed analysis of functional magnetic resonance imaging in the frontal language area: A comparative study with extraoperative electrocortical stimulation. Neurosurgery.

[CR51] Kunii N, Kawai K, Kamada K, Ota T, Saito N (2014). The significance of parahippocampal high gamma activity for memory preservation in surgical treatment of atypical temporal lobe epilepsy. Epilepsia.

[CR53] Lega BC, Jacobs J, Kahana M (2012). Human hippocampal theta oscillations and the formation of episodic memories. Hippocampus.

[CR52] Lega B, Burke J, Jacobs J, Kahana MJ (2016). Slow-Theta-to-Gamma phase–amplitude coupling in human Hippocampus supports the formation of New Episodic Memories. Cerebral Cortex.

[CR54] Lin JJ, Rugg MD, Das S, Stein J, Rizzuto DS, Kahana MJ, Lega BC (2017). Theta band power increases in the posterior hippocampus predict successful episodic memory encoding in humans. Hippocampus.

[CR55] Lofthouse N, Arnold LE, Hersch S, Hurt E, DeBeus R (2012). A review of Neurofeedback Treatment for Pediatric ADHD. Journal of Attention Disorders.

[CR57] Long NM, Kahana MJ (2015). Successful memory formation is driven by contextual encoding in the core memory network. Neuroimage.

[CR56] Long NM, Burke JF, Kahana MJ (2014). Subsequent memory effect in intracranial and scalp EEG. Neuroimage.

[CR58] Mathiak KA, Alawi EM, Koush Y, Dyck M, Cordes JS, Gaber TJ, Zepf FD, Palomero-Gallagher N, Sarkheil P, Bergert S, Zvyagintsev M, Mathiak K (2015). Social reward improves the voluntary control over localized brain activity in fMRI-based neurofeedback training. Frontiers in Behavioral Neuroscience.

[CR60] Mihara T, Inoue Y, Watanabe Y, Matsuda K, Tottori T, Hiyoshi T, Kubota Y, Yagi K, Seino M (1994). Improvement of Quality-of-life following resective surgery for temporal lobe Epilepsy: Results of patient and family assessments. Psychiatry and Clinical Neurosciences.

[CR59] Mihara, T., Inoue, Y., Matsuda, K., Tottori, T., Otsubo, T., Watanabe, Y., Hiyoshi, T., Kubota, Y., Yagi, K., & Seino, M. (1996). Recommendation of Early Surgery from the Viewpoint of Daily Quality of Life. *Epilepsia*, *37*(SUPPL. 3), 33–36. 10.1111/J.1528-1157.1996.TB01817.X 10.1111/j.1528-1157.1996.tb01817.x 8681909

[CR62] Miller NE (1969). Learning of visceral and glandular responses. Science.

[CR61] Miller J, Watrous AJ, Tsitsiklis M, Lee SA, Sheth SA, Schevon CA, Smith EH, Sperling MR, Sharan A, Asadi-Pooya AA, Worrell GA, Meisenhelter S, Inman CS, Davis KA, Lega B, Wanda PA, Das SR, Stein JM, Gorniak R, Jacobs J (2018). Lateralized hippocampal oscillations underlie distinct aspects of human spatial memory and navigation. Nature Communications.

[CR63] Monseigne, T., Lotte, F., Bioulac, S., Philip, P., Micoulaud-Franchi, J. A., & Bioulac, S. (2019). Design and preliminary study of a neurofeedback protocol to self-regulate an EEG marker of drowsiness. *Proceedings of The 8th Graz Brain-Computer Interface Conference*, *Austria*, 200–205. 10.3217/978-3-85125-682-6-37

[CR64] Mormann F, Fell J, Axmacher N, Weber B, Lehnertz K, Elger CE, Fernández G (2005). Phase/amplitude reset and theta–gamma interaction in the human medial temporal lobe during a continuous word recognition memory task. Hippocampus.

[CR65] Mullen TR, Kothe CAE, Chi YM, Ojeda A, Kerth T, Makeig S, Jung TP, Cauwenberghs G (2015). Real-time neuroimaging and cognitive monitoring using wearable dry EEG. IEEE Transactions on Biomedical Engineering.

[CR1] National Institute for Japanese Language and Linguistics (2019, November 26). *WLSP-familiarity (ver. 1.0).* Retrieved February 20, 2020, from https://github.com/masayu-a/WLSP-familiarity

[CR66] Oblak EF, Lewis-Peacock JA, Sulzer JS (2017). Self-regulation strategy, feedback timing and hemodynamic properties modulate learning in a simulated fMRI neurofeedback environment. PLOS Computational Biology.

[CR67] Osipova D, Takashima A, Oostenveld R, Fernández G, Maris E, Jensen O (2006). Theta and Gamma Oscillations Predict Encoding and Retrieval of declarative memory. Journal Of Neuroscience.

[CR68] Parvizi J, Kastner S (2018). Promises and limitations of human intracranial electroencephalography. Nature Neuroscience.

[CR70] Reiner M, Rozengurt R, Barnea A (2014). Better than sleep: Theta neurofeedback training accelerates memory consolidation. Biological Psychology.

[CR69] Reiner M, Lev DD, Rosen A (2018). Theta Neurofeedback Effects on Motor Memory consolidation and performance accuracy: An Apparent Paradox?. Neuroscience.

[CR71] Reis, J., Portugal, A. M., Fernandes, L., Afonso, N., Pereira, M., Sousa, N., & Dias, N. S. (2016). An alpha and theta intensive and short neurofeedback protocol for healthy aging working-memory training. *Frontiers in Aging Neuroscience*, *8*(157), 10.3389/fnagi.2016.00157 10.3389/fnagi.2016.00157 PMC4936375 27458369

[CR72] Richardson, M. P., Strange, B. A., Duncan, J. S., & Dolan, R. J. (2003). Preserved verbal memory function in left medial temporal pathology involves reorganisation of function to right medial temporal lobe. *NeuroImage*, *20*(SUPPL. 1), S112–S119. 10.1016/J.NEUROIMAGE.2003.09.008 10.1016/j.neuroimage.2003.09.008 14597304

[CR73] Rozengurt R, Shtoots L, Sheriff A, Sadka O, Levy DA (2017). Enhancing early consolidation of human episodic memory by theta EEG neurofeedback. Neurobiology of Learning and Memory.

[CR74] Schoenberg PLA, David AS (2014). Biofeedback for psychiatric disorders: A systematic review. Applied Psychophysiology Biofeedback.

[CR75] Sederberg PB, Kahana MJ, Howard MW, Donner EJ, Madsen JR (2003). Theta and Gamma Oscillations during Encoding Predict subsequent Recall. Journal of Neuroscience.

[CR77] Sederberg PB, Schulze-Bonhage A, Madsen JR, Bromfield EB, McCarthy DC, Brandt A, Tully MS, Kahana MJ (2007). Hippocampal and neocortical Gamma Oscillations Predict memory formation in humans. Cerebral Cortex.

[CR76] Sederberg PB, Miller JF, Howard MW, Kahana MJ (2010). The temporal contiguity effect predicts episodic memory performance. Memory & Cognition.

[CR78] Sherlin LH, Arns M, Lubar J, Heinrich H, Kerson C, Strehl U, Sterman MB (2011). Neurofeedback and Basic Learning Theory: Implications for Research and Practice. Journal of Neurotherapy.

[CR79] Solomon, E. A., Lega, B. C., Sperling, M. R., & Kahana, M. J. (2019a). Hippocampal theta codes for distances in semantic and temporal spaces. Proceedings of the National Academy of Sciences, 116(48), 24343–24352. https://doi.org/10.1073/pnas.1906729116. 10.1073/pnas.1906729116 PMC6883851 31723043

[CR80] Solomon EA, Stein JM, Das S, Gorniak R, Sperling MR, Worrell G, Inman CS, Tan RJ, Jobst BC, Rizzuto DS, Kahana MJ (2019). Dynamic Theta Networks in the human medial temporal lobe support episodic memory. Current Biology.

[CR81] Squire, L. R., & Zola-Morgan, S. (1991). The medial temporal lobe memory system. In *Science* (Vol. 253, Issue 5026, pp. 1380–1386). 10.1126/science.1896849 10.1126/science.1896849 1896849

[CR82] Staudigl T, Hanslmayr S (2013). Theta Oscillations at Encoding mediate the context-dependent nature of human episodic memory. Current Biology.

[CR83] Sterman MB, Wyrwicka W, Roth S (1969). Electrophysiological correlates and neural substrates of alimentary behavior in the cat. Annals of the New York Academy of Sciences.

[CR84] Sulzer J, Haller S, Scharnowski F, Weiskopf N, Birbaumer N, Blefari ML, Bruehl AB, Cohen LG, deCharms RC, Gassert R, Goebel R, Herwig U, LaConte S, Linden D, Luft A, Seifritz E, Sitaram R (2013). Real-time fMRI neurofeedback: Progress and challenges. Neuroimage.

[CR85] Takabatake K, Kunii N, Nakatomi H, Shimada S, Yanai K, Takasago M, Saito N (2021). Musical auditory alpha Wave Neurofeedback: Validation and cognitive perspectives. Applied Psychophysiology Biofeedback.

[CR86] Tani N, Kishima H, Khoo HM, Yanagisawa T, Oshino S, Maruo T, Hosomi K, Hirata M, Kazui H, Nomura KT, Aly MM, Kato A, Yoshimine T (2016). Electrical stimulation of the parahippocampal gyrus for prediction of posthippocampectomy verbal memory decline. Journal of Neurosurgery.

[CR87] Trenerry MR, Jack CR, Ivnik RJ, Sharbrough FW, Cascino GD, Hirschorn KA, Marsh WR, Kelly PJ, Meyer FB (1993). MRI hippocampal volumes and memory function before and after temporal lobectomy. Neurology.

[CR88] Tseng YH, Tamura K, Okamoto T (2021). Neurofeedback training improves episodic and semantic long-term memory performance. Scientific Reports.

[CR89] Wang JR, Hsieh S (2013). Neurofeedback training improves attention and working memory performance. Clinical Neurophysiology.

[CR90] World Health Organization (2019). *Epilepsy: a public health imperative: summary*. https://apps.who.int/iris/bitstream/handle/10665/325440/WHO-MSD-MER-19.2-eng.pdf

[CR91] Yamin HG, Gazit T, Tchemodanov N, Raz G, Jackont G, Charles F, Fried I, Hendler T, Cavazza M (2017). Depth electrode neurofeedback with a virtual reality interface. Brain-Computer Interfaces.

[CR92] Zilverstand A, Sorger B, Sarkheil P, Goebel R (2015). fMRI neurofeedback facilitates anxiety regulation in females with spider phobia. Frontiers in Behavioral Neuroscience.

